# The frequency of postoperative hypoglycemia after pheochromocytoma surgery is decreasing

**DOI:** 10.1002/wjs.12368

**Published:** 2024-10-09

**Authors:** Yuki Yamanashi, Yusaku Yoshida, Tomoyoshi Nakai, Juro Yanagida, Yoko Omi, Kiyomi Horiuchi

**Affiliations:** ^1^ Department of Endocrine Surgery Tokyo Women's Medical University Tokyo Japan

**Keywords:** alpha blockade, hypoglycemia, pheochromocytoma

## Abstract

**Background:**

Hypoglycemia after pheochromocytoma resection is one of the most common postoperative complications, with a reported incidence of 12%–43%. In recent years, we have rarely experienced postoperative hypoglycemia after pheochromocytoma surgery at our institution. We reviewed our own experience and examined factors associated with postoperative hypoglycemia in pheochromocytoma patients.

**Methods:**

We collected and retrospectively reviewed medical information from 53 patients with pheochromocytoma who underwent initial surgery in our department between 1996 and 2022, who did not receive steroids in the perioperative period and received the same alpha‐blocker preoperatively. Subjects were divided into two groups by the midpoint of the study period: Group 1 (G1), 1996–2009; and Group 2 (G2), 2010–2022. The two groups were compared.

**Results:**

Hypoglycemia occurred significantly less often in G2 (0 patients, 0%) than in G1 (7 patients, 28%; *p* = 0.003). Preoperative diabetes was significantly less frequent in G2 (2 patient, 7.1%) than in G1 (8 patients, 32%; *p* = 0.03). Preoperative alpha‐blocker dosage was significantly higher in G2 than in G1 (*p* = 0.04). Multivariate logistic regression analysis showed that only alpha‐blockers dosage was significantly associated with the occurrence of postoperative hypoglycemia (*p* = 0.004).

**Conclusion:**

The current study suggest that the alpha‐blocker dosage might be related to the lower incidence of postoperative hypoglycemia in Pheochromocytoma patients.

## 1 INTRODUCTION

Pheochromocytomas are neuroendocrine tumors that arise from catecholamine‐producing chromophilic cells in the adrenal medulla. These tumors produce catecholamines, the excess of which causes myocardial damage, heart failure, ischemic heart disease, hypertension, and hyperglycemia, in turn leading to cerebral and cardiovascular events.^1^ In addition, the alpha and beta effects of catecholamines complicate metabolic abnormalities including impaired glucose tolerance.^2^, ^3^, ^4^, ^5^

Surgical removal of the tumor is the first‐line treatment for pheochromocytoma.^6^ However, strict circulatory control is required after surgery because the rapid decrease in catecholamine secretion resulting from tumor removal can cause hypotension and hemodynamic compromise.^1^ ^,^ ^7^, ^8^, ^9^ At the same time, release of the insulin resistance and suppression of the insulin secretion caused by excessive catecholamine secretion by pheochromocytoma can lead to hypoglycemia.^3^, ^4^, ^5^ ^,^ ^10^, ^11^, ^12^ Prolonged hypoglycemia can induce central nervous system complications such as hypoglycemic encephalopathy and coma, requiring careful management as with blood pressure management.^13^, ^14^ Although this postoperative hypoglycemia has been reported to occur at a rate of 12%–43%,^15^, ^16^, ^17^, ^18^ we felt that in recent years we had rarely experienced postoperative hypoglycemia after pheochromocytoma surgery at our own institution. We therefore analyzed our own case series to observe changes in the incidence of postoperative hypoglycemia in pheochromocytoma over time and to examine factors associated with the occurrence of hypoglycemia.

## 2 MATERIALS AND METHODS

Participants comprised 190 patients who underwent surgery for pheochromocytoma at the Department of Endocrine Surgery, Tokyo Women's Medical University Hospital between January 1996 and December 2022. Inclusion criteria were patients who underwent initial surgery, whose blood pressure was controlled with the same alpha‐blocker (doxazosin), and for whom data were available for preoperative 24‐h urine metanephrine (MN) fraction, operation time as well as preoperative glucose tolerance index. This index includes Homeostatic Model Assessment for *β* cell function (HOMA‐β) and Homeostatic Model Assessment for insulin resistance (HOMA‐IR) calculated using the 75‐g oral glucose tolerance test (OGTT).^19^

Exclusion criteria included perioperative administration of steroids. Patients using other types of antihypertensive drugs such as *β*‐blockers and calcium channel blockers were not included in the study.

We usually use doxazosin, which is α1‐selective. Dosage varies among outpatient physicians, but our institution currently starts at approximately 1–3 mg/day and increases the dosage by approximately 2 mg every week, with a target of ≥12 mg/day for patients with hypertension or clinical symptoms of catecholamine excess, and 10 mg/day for patients without hypertension or catecholamine excess. All patients were managed in the intensive care unit postoperatively, and were given maintenance infusions with glucose concentrations of 4.3%–10%, with blood glucose measured hourly.

From the medical records, plasma catecholamine fraction measured 2 days before surgery, 24‐h urine MN fraction and results of a 75‐g OGTT test performed the day before surgery were extracted.

## 3 EVALUATION OF HYPOGLYCEMIA

The observation period was from the time of admission to the postoperative intensive care unit until discharge the morning of the next day, and a blood glucose reading of ≤70 mg/dl taken every hour was considered to represent the onset of hypoglycemia.

The primary outcome of this study was to observe changes in the incidence of postoperative hypoglycemia in pheochromocytoma over time, and the secondary outcome was to examine factors that cause hypoglycemia.

## 4 STATISTICAL ANALYSIS

Patients were divided into two groups according to the midpoint of the study period: Group 1 (G1), 1996–2009; and Group 2 (G2), 2010–2022.

First, the frequency of postoperative hypoglycemia was compared between groups. The groups were then compared and analyzed for significant differences in the following 9 factors: presence of preoperative diabetes; HOMA‐β; HOMA‐IR; 24‐h urine MN; 24‐h urine normetanephrine (NMN); 24‐h total urine MN fraction; doxazosin dosage at surgery; tumor size; surgical technique (open or laparoscopic); and operation time.

Groups with and without postoperative hypoglycemia were also compared, and items with significant differences among the above 10 items were analyzed.

The presence of diabetes mentioned in this study was defined as patients who had already started treatment with insulin or oral hypoglycemic agents before surgery or who fit the condition for diabetic type on the OGTT test after admission to the hospital.

For both categorical variables, the χ^2^ test was performed and significant differences were calculated with Fisher's exact test. For continuous variables, Student's *t*‐test was used to calculate significant differences if equal variance was found, and Welch's *t*‐test was used to calculate significant differences if not found.

Finally, we performed multivariate logistic regression analysis with the presence of preoperative diabetes mellitus, 24‐h urine MN, 24‐h urine NMN, doxazosin dosage, and operation time, which we expected to be strongly related to the occurrence of hypoglycemia. Odds ratios were calculated as the ratio of the occurrence of postoperative hypoglycemia to the absence of hypoglycemia. For continuous variables, odds ratios were calculated per 1‐mg/day change in urinary metanephrine stores, per 1‐mg change in alpha‐blocker dosage, and per 1‐mm change in tumor diameter.

In all cases, differences were considered to be significant for values of *P* < 0.05. All statistical analyses were performed using JMPpro17 (SAS Institute, Inc). This clinical study was approved by the ethics committee at Tokyo Women's Medical University (approval no. 2024‐0016).

## 5 RESULTS

**FIGURE 1:**
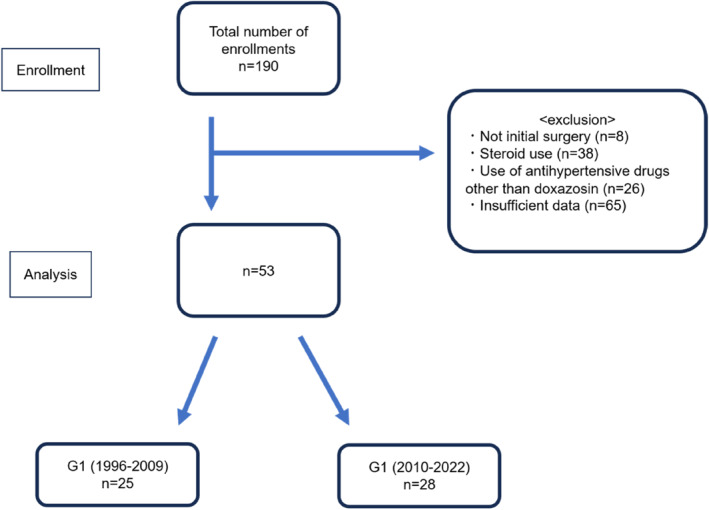
Consort flow diagram. Of the 190 patients concerned, 53 were included in the study, excluding patients who were not undergoing initial surgery, who were using alpha‐blockers other than doxazosin, who were using steroids, and who had insufficient data. Patients were classified as G1 or G2 according to age.

Participation criteria were met in 53 cases (Figure 1), comprising 27 men and 26 women. Median age was 44 years (range, 18–82 years). G1 comprised 13 men and 12 women, with a median age of 45 years (range, 18–82 years). G2 comprised 14 men and 14 women, with a median age of 44 years (range, 21–81 years). Patient characteristics and preoperative endocrinological test results are shown in Table 1. Values for each group are expressed as median and range. Sex, age, and postoperative supplemental fluid concentration did not differ significantly between groups (*p* = 1.00, *p* = 0.90, *p* = 0.31).

**TABLE 1:** Patient characteristics.

	Total (*n* = 50)	G1 (*n* = 26)	G2 (*n* = 24)	*p* value
Sex, male/female	27/26	13/12	14/14	1.00
Age, years (range)	44 (18–82)	45 (18–82)	44 (21–81)	0.90
Postoperative hypoglycemia, *n* (%)	7 (13)	7 (28)	0 (0)	0.003
Postoperative infusion glucose level, %, median (range)	7.5 (4.3–10)	7.5 (4.3–10)	7.5 (7.5–7.5)	0.31
Diabetes mellitus, *n* (%)	10 (19)	8 (32)	2 (7.1)	0.03
HOMA‐β, %, median (range)	57.8 (6.5–176)	52.5 (6.5–176)	59.6 (14.9–162.8)	0.78
HOMA‐IR, %, median (range)	1.6 (0.5–16.3)	1.2 (0.5–16.3)	1.75 (0.5–5.3)	0.73
24‐h Urine metanephrine, mg/day, median (range)	0.66 (0.06–11.57)	0.57 (0.06–11.57)	0.72 (0.08–10.4)	0.60
24‐h Urine normetanephrine, mg/day, median (range)	1.39 (0.21–12.84)	2.1 (0.25–12.84)	0.88 (0.21–12.4)	0.06
24‐h Total urine metanephrine, mg/day, median (range)	2.06 (0.43–15)	3.25 (0.81–13.56)	1.6 (0.43–15)	0.33
α‐blocker dose at surgery, mg, median (range)	10 (4–16)	8 (4–16)	12 (6–14)	0.04
Tumor size, mm, median (range)	42 (19–100)	45 (20–78)	39 (19–100)	0.29
Open/Laparoscopic	8/45	7/18	1/27	0.02
Operation time (min)	196 (85–405)	191 (87–405)	196.5 (85–368)	0.31

A comparison of groups G1 and G2 showed that hypoglycemia was significantly less frequent in G2 (0 patients, 0%) than in G1 (7 patients, 28%; *p* = 0.003). The frequency of preoperative diabetes was significantly lower in G2 (2 patient, 7.1%) than in G1 (8 patients, 32%; *p* = 0.03). In addition, none of the seven cases experienced in our department had symptoms due to hypoglycemia.

Further, the dosage of alpha‐blockers was significantly higher in G2 (*p* = 0.04) and differences in surgical technique were also significant, with laparoscopic procedures more common in G2 (*p* = 0.02).

No other significant differences were apparent between groups for other items (Table 1).

Analysis between groups according to presence or absence of hypoglycemia showed that HOMA‐IR and the dosage of alpha‐blockers are differed significantly. The dosage and HOMA‐IR were significantly higher in the non‐postoperative hypoglycemia group (*p* = 0.005, *p* = 0.01) (Table 2).

**TABLE 2:** Comparison between groups according to presence of hypoglycemia.

	Postoperative hypoglycemia (*n* = 7)	No postoperative hypoglycemia (*n* = 43)	*p* value
Preoperative diabetes, *n* (%)	2 (28.5%)	8 (17.4%)	0.60
HOMA‐β, %, median (range)	59.6 (6.5–120)	56.6 (9–176)	0.80
HOMA‐IR, %, median (range)	1 (0.5–2.1)	1.7 (0.5–16.3)	0.01
24‐h Urine metanephrine, mg/day, median (range)	0.77 (0.11–11.57)	0.64 (0.06–10.4)	0.43
24‐h Urine normetanephrine, mg/day, median (range)	2.8 (0.67–7.9)	1.29 (0.21–12.8)	0.52
24‐h Total urine metanephrine, mg/day, median (range)	3.8 (0.82–12.23)	1.92 (0.43–15)	0.17
α‐blocker dose at the surgery, mg, median (range)	6 (5–12)	10 (4–16)	0.005
Tumor size, mm, median (range)	55 (20–78)	41 (19–100)	0.62
Open/Laparoscopic	1/6	7/39	1.00
Operation time (min)	259 (87–339)	193.5 (85–405)	0.56

Logistic regression analysis employed the presence of preoperative diabetes mellitus, 24‐h urine MN, 24‐h urine NMN, doxazosin dosage and operation time as explanatory variables. Only the dosage of alpha blockers was associated with postoperative hypoglycemia (*p* = 0.004; odds ratio 0.47, 95% CI 0.28–0.79), with no associations between presence of preoperative diabetes mellitus, 24‐h urine MN, 24‐h urine NMN, and operation time (Table 3).

**TABLE 3:** Logistic regression analysis.

Parameter estimation	Estimated value	Standard error	Chi‐square	*p* value	Odds ratio^a^ (95% CI)
Intercept	1.57	2.29	0.47	0.49	
Preoperative diabetes mellitus	0.29	0.82	0.12	0.72	1.78 (0.07–44.16)
24‐h Urine metanephrine	0.35	0.21	2.78	0.09	1.41 (0.94–2.13)^b^
24‐h Urine normetanephrine	0.26	0.19	1.19	0.15	1.30 (0.90–1.88)^c^
α‐blocker dose at surgery	−0.74	0.26	8.13	0.004	0.47 (0.28–0.79)^d^
Operation time	0.006	0.007	0.79	0.37	1.00 (0.99–1.01)^e^

^a^ hypoglycemia occur/not occur.

^b^ 24‐h urine metanephrine per 1 mg/day increase.

^c^ 24‐h urine normetanephrine per 1 mg/day increase.

^d^ *α*‐blocker dose per 1 mg increase.

^e^ tumor size per 1 mm increase.

## 6 DISCUSSION

This novel report examined our recent perception of a decrease in the incidence of postoperative hypoglycemia. The results confirmed that there were fewer diabetic patients in G2, they tended to receive higher dosages of doxazosin, and more notably, G2 showed no cases of postoperative hypoglycemia. Multivariate analysis showed that only the dosage of alpha‐blocker correlated significantly with the occurrence of postoperative hypoglycemia.

Preoperative administration of alpha‐blockers in patients with pheochromocytoma is considered important for perioperative hemodynamic management and has been widely indicated since 1956.^20^

The pheochromocytoma and paraganglioma (PPGL) practice guidelines by the Japan Endocrine Society published in 2018 recommend preoperative alpha‐blocker administration, as the perioperative mortality rate has decreased to less than 3% with the introduction of alpha‐blockers.^21^, ^22^ In Japan, only selective alpha 1 blocker is covered by insurance, and the guidelines recommend starting with doxazosin at 1–2 mg/day and titrating every 2–3 days to achieve normotension. To compensate for the decrease in circulating blood volume, the suggestion has been made, without scientific evidence, that salt intake be increased after day 3 of alpha‐blocker administration, depending on the degree of hypotension, and that 1–2 L of saline solution be infused in the evening on the day before surgery.^22^

In addition, the Endocrine Society guidelines published in 2014 recommended 7–14 days of preoperative alpha‐blockade for functional pheochromocytoma and PPGL, which is associated with lower preoperative diastolic blood pressure, lower intraoperative pulse rate and postoperative hemodynamic recovery.^23^ The North American Neuroendocrine Tumor Society guidelines published in 2010 state that all patients with PPGL, including normotensive patients, should receive alpha‐blockers for a minimum of 10–14 days, since appropriate alpha‐blocker administration reduces the incidence of perioperative complications.^24^ However, no detailed descriptions of how to increase the drug dosage or the preferable maximum dosage have been reported. The optimal dosage may be determined by facility‐specific criteria, which are believed to be approximately a sitting systolic blood pressure of 130/80 mmHg, a sitting pulse rate of 60–70 beats/min, and a standing pulse rate of 70–80 beats/min.^25^ In our department, the starting dose of doxazosin is 1–3 mg/day, although there are some differences depending on the attending physician. In recent years, based on Japanese guidelines, we have been aiming a target dose of 12 mg or more before surgery in cases where there is hypertension or excessive catecholamine production, and 10 mg in cases where there is no hypertension or excessive catecholamine production, within the range that does not cause orthostatic hypotension. The dosage is also increased to the patient's blood pressure less than 140/90 mmHg before surgery, and their pulse rate between 60 and 70 beats per minute when sitting, or between 70 and 80 beats per minute when standing.

In recent years, however, many reports have advocated the omission of preoperative alpha‐blocker administration because of the lack of significant differences in intraoperative hemodynamic changes, postoperative complications, and mortality rates between groups with and without preoperative alpha‐blockers.^26^, ^27^, ^28^, ^29^, ^30^ A systematic review of randomized and nonrandomized controlled studies that collected these reports and evaluated preoperative alpha‐blockade for PPGL surgery in adults found that preoperative alpha‐blockade had no beneficial effect on intraoperative hemodynamics or perioperative complications or mortality compared with the non‐treated group. However, none of the 15 articles reviewed mentioned preoperative alpha‐blocker administration or the occurrence of postoperative hypoglycemia.^31^

A multicenter review of perioperative management and complications of pheochromocytoma reported three cases of hypoglycemia in the group that received alpha‐blockers compared to no cases of hypoglycemia in the group that did not receive alpha‐blockers as pretreatment.^32^ The frequency of hypoglycemia in this study appears to be consistent with our report.

However, in our study, all patients were administered alpha‐blockers, so the populations are different. For this reason, it seems that the two reports cannot be compared simply.

In addition, there is no mention of the amount of glucose infusion or the amount of perioperative steroid administration, and it is possible that these may have affected the incidence of postoperative hypoglycemia.

As for the cause of glucose intolerance in patients with pheochromocytoma, α2 receptors are mainly involved in decreased insulin secretion, while α1, β2, and β3 receptors in each tissue are associated with insulin resistance. In addition, increased hepatic glycogenesis and glycogenolysis due to increased levels of free fatty acids via β2 and α1 receptors, and decreased glucose uptake in skeletal muscle via mechanisms of decreased glucose uptake via β1 and α1 receptors have been proposed.^3^, ^4^, ^5^ ^,^ ^10^ ^,^ ^12^ ^,^ ^15^ ^,^ ^33^ Many reports have found that postoperatively, a rapid decrease in catecholamines releases *α* and *β* stimulation, resulting in decreased blood glucose levels due to insulin recoil secretion, decreased gluconeogenesis, and increased insulin sensitivity.^5^ ^,^ ^10^, ^11^, ^12^, ^13^, ^14^, ^15^ In addition, when the half‐life of doxazosin is taken together with reports of the favorable time for postoperative hypoglycemia in post‐pheochromocytoma patients, hypoglycemic attacks are expected to occur at least 2–4 h after surgery.^11^ ^,^ ^15^ We speculated that adequate preoperative administration of doxazosin may postoperatively reduce both the rapid change in alpha‐receptor action and the occurrence of hypoglycemia.^5^ ^,^ ^15^

In a previous study on the occurrence of hypoglycemia after pheochromocytoma surgery, Akiba et al. investigated the causative factors and reported that preoperative epinephrine hypersecretion and the presence of diabetes mellitus or glucose intolerance were associated with the occurrence of hypoglycemia.^5^ Also in 2014, chen et al. reported that epinephrine significant type and long surgery time are associated factors for postoperative hypoglycemia.^17^ However, with regard to the administration of alpha‐blockers, there was mention of their types, but no mention of differences in dosage.

In the current analysis, we found that a selective alpha 1 blocker (doxazosin) may be significantly associated with a reduced occurrence of postoperative hypoglycemia after pheochromocytoma surgery. This is a new finding compared to previous reports because our study included patients who received the same *α*‐blocker, allowing more precise analysis of the involvement of alpha‐blockers in postoperative hypoglycemia.

Limitations in this study were as follows. First, patients on insulin therapy or oral hypoglycemic medications preoperatively were analyzed as diabetics. After admission, medication was continued until preoperatively, so HOMA is only a reference value for such patients. Measurement of C‐peptide is desirable, but since we did not measure this value, the original measurement of impaired glucose tolerance may have been inadequate. Next, we did not examine differences in anesthesia and surgical methods in this study. Improvements in anesthesia techniques, drugs, and surgical technique may also have contributed to the decrease in hypoglycemia. Further, this study included only cases in which doxazosin was administered as an alpha‐blocker. Since the use of non‐selective alpha‐blockers is predominant in other countries, not necessarily all patients with pheochromocytoma will be eligible for the results of this study. Finally, this study only represents an analysis of findings from our institution.

However, based on this study, adequate preoperative administration of alpha‐blockers appears desirable, and omission of alpha‐blocker administration, which has been advocated in recent years,^26^, ^27^, ^28^, ^29^, ^30^ should be judged with caution. Even though the frequency of hypoglycemia is decreasing, stopping regular blood glucose monitoring to avoid missing this finding may not be advisable.

## 7 CONCLUSION

In recent years, postoperative hypoglycemia among pheochromocytoma patients has become less common and diabetic patients have also been accounting for a smaller proportion. Postoperative hypoglycemia have the potential to be correlated with the dosage of alpha‐blockers. Preoperative administration of alpha‐blockers to patients with pheochromocytoma may prevent postoperative hypoglycemia.

## AUTHOR CONTRIBUTIONS

**Yuki Yamanashi**: Conceptualization; data curation; formal analysis; investigation; methodology; resources. **Yusaku Yoshida**: Conceptualization; data curation; project administration; resources; writing – review & editing. **Tomoyoshi Nakai**: Data curation; resources. **Juro Yanagida**: Data curation; resources. **Yoko Omi**: Data curation; resources. **Kiyomi Horiuchi**: Conceptualization; data curation; project administration; resources; writing – review & editing.

## CONFLICT OF INTEREST STATEMENT

None.

## ETHICS STATEMENT

This clinical study was approved by the ethics committee at Tokyo Women's Medical University (approval no. 2024‐0016).
